# Invasive mole in a perimenopausal woman with lung and vaginal metastases: A case report

**DOI:** 10.1002/ccr3.2386

**Published:** 2019-09-30

**Authors:** Cristina Martínez Leocadio, José García Villayzán, Jesús García‐Foncillas López, Franklin Idrovo, Javier Plaza Arranz, Manuel Albi González

**Affiliations:** ^1^ Department of Obstetrics and Gynecology, Fundación Jiménez Díaz University Hospital Madrid Spain; ^2^ Department of Oncology, Fundación Jiménez Díaz University Hospital Madrid Spain; ^3^ Department of Anatomic Pathology, Fundación Jiménez Díaz University Hospital Madrid Spain

**Keywords:** gestational trophoblastic neoplasia, hysterectomy, invasive mole, metastases, perimenopausal, trophoblastic disease

## Abstract

Gestational trophoblastic disease can result in serious complications and disease progression. Therefore, follow‐up of such patients is essential for early detection of malignant trophoblastic tumors and to reduce mortality rate. Primary treatment is chemotherapy but hysterectomy should be considered in patients who have uncontrollable hemorrhage and hemodynamic instability.

## Graphical abstract

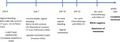

## 1 INTRODUCTION

**Figure 1:**
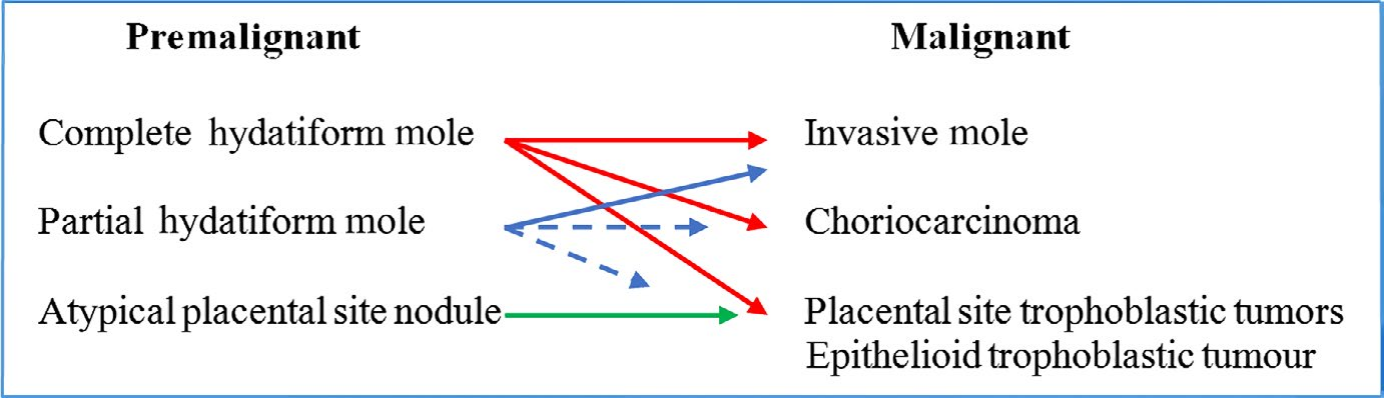
Gestational trophoblastic disease spectrum

Gestational trophoblastic disease (GTD) is a spectrum of cellular proliferations arising from the placental villous trophoblast.^1^ It encompasses three premalignant conditions: partial hydatidiform mole (PHM), complete hydatidiform mole (CHM), and atypical placental site nodule. Moreover, GTD can progress to three malignant gestational trophoblastic neoplasias (GTN). GTN are classified histologically into three distinct subgroups: invasive mole, choriocarcinoma (CC), and the very rare placental site trophoblastic tumor (PSTT)/epithelioid trophoblastic tumor (ETT).^2^, ^3^ (Figure 1).

Hydatidiform mole incidence is of 1 in 591 pregnancies and 1 in 714 live births in the developed world. The two most important risk factors for the development of a molar pregnancy are age and history of previous pregnancies. Extreme maternal age significantly increases the risk of developing a molar pregnancy, especially complete mole. The risk also increases in women over 40 years old, being 7‐8 times greater than in women between 20 and 35 years old.^4^ The risk of developing a new molar pregnancy increases from 1%‐2% in patients with one previous mole, to 15%‐20% in patients with two previous molar pregnancies.^5^

Invasive mole diagnosis is based on the presence of a hydatidiform mole with myometrial, blood or lymphatic invasion or the presence of distant metastases and an abnormal HCG regression. The progression of a hydatidiform mole to an invasive mole is about 10%‐17%, being more frequent in the case of complete mole.^4^

The most frequent clinical presentation of invasive mole includes persistent or heavy vaginal bleeding after evacuation of molar pregnancy. When invasive mole is either suspected or confirmed histopathologically, to perform a thoracoabdominopelvic CT and cerebral MRI is mandatory. In rare cases, metastases occurred most frequently in the following sites: the lung (80%) followed by the vagina (30%), the liver (10%), and the brain (10%).^6^

Chemotherapy is the primary treatment of choice for invasive mole. FIGO prognostic scoring system determines the risk of resistance to methotrexate. The regimen for the low‐risk patients (FIGO score < 7) is methotrexate 50 mg IM on days 1, 3, 5, and 7 alternated with folates 15 mg PO on days 2, 4, and 6. In patients with poor response to methotrexate (serum and BHCH increase or plateau), actinomycin D should be added or multidrug therapy, which has a complete remission rate close to 100%. In high‐risk patients (FIGO score ≥ 7) the polychemotherapeutic treatment according to the EMA‐CO regimen (Etoposide + Metotrexate + Actinomycin D alternating with Cyclophosphamide + Vincristine) is the preferred option. BHCG remission and normalization with the EMA‐CO regimen is 98%. In case of poor response to this treatment, EMA‐EP or BEP (Bleomycin + Etoposide + Cisplatin) should be considered.^7^, ^8^ In WHO score system, risk score (5‐6) and clinicopathologic diagnosis of choriocarcinoma are both associated with an increased risk of resistance to single‐agent chemotherapy. Lowering the threshold for the use of multiple‐agent chemotherapy in these otherwise low‐risk patients can be considered.^2^

Finally, if myometrial invasion is suspected, uterine curettage and endometrial biopsy are contraindicated^7^ due to the risk of uterine perforation. The surgical resection of metastases in high‐risk patients is not recommended, save in the case of residual lesions in patients resistant to chemotherapy. Biopsy of metastases is not recommended due to the risk of hemorrhage.^8^

The risk of recurrence of chemotherapy‐treated trophoblastic disease is 3%.^8^ Therefore, a follow‐up of at least 12 months with BHCG monthly determination is recommended, starting from the first two consecutive BHCG negative weeks.^7^, ^8^ Hormonal contraception is indicated during this period.^7^

## 2 CASE PRESENTATION

**Figure 2:**
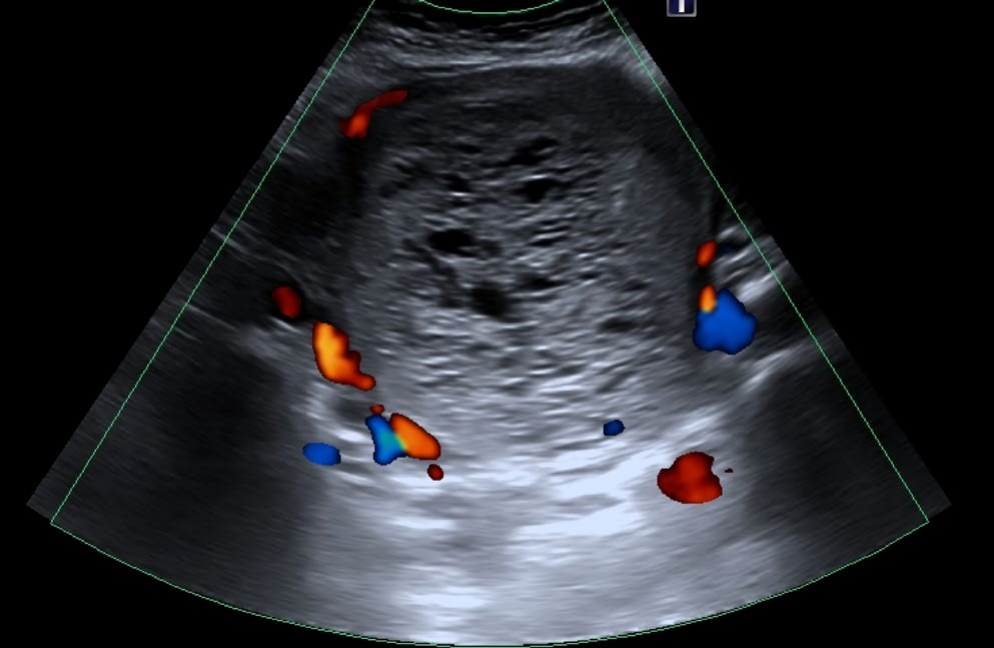
Abdominopelvic US: Vesicular pattern of multiple echoes

**Figure 3:**
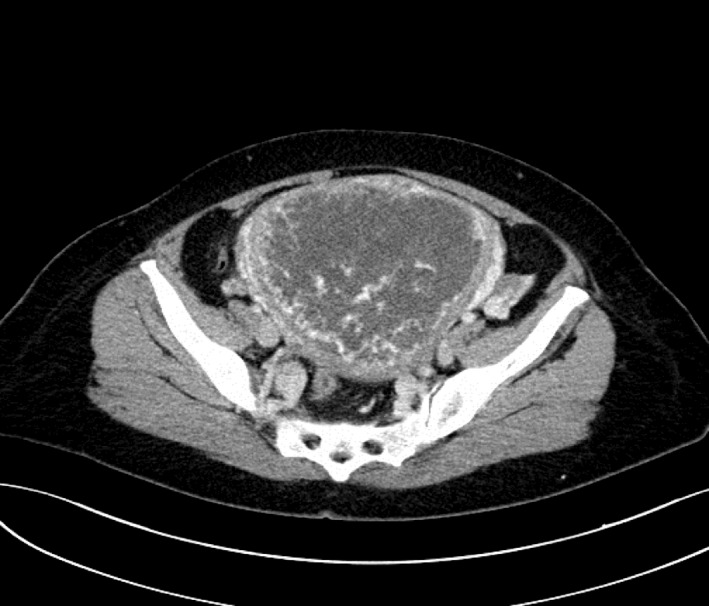
Thoracoabdominopelvic CT scan: Pelvic mass of 14 cm

A 53‐year‐old woman, with an obstetric history of 7 pregnancies (5 vaginal births and 2 abortions) with the last date of menstruation 6 months ago. She was presented to the hospital with severe vaginal bleeding and abdominal pain. Days before, she had suffered an episode of hematemesis and epigastralgia, and she was assessed by the Gastrointestinal Department, who performed a gastroscopy with normal biopsies, and the endoscopic exploration showed erosive lesions in relation to NSAID. On gynecologic examination, there were no vulvar, vaginal, or cervical lesions, and an enlarged uterus was noted. An abdominal ultrasonography (Figure 2) and thoracoabdominopelvic CT scan (Figure 3) were performed, demonstrating a pelvic mass of 14 cm suggestive of trophoblastic disease and two nonspecific millimetric pulmonary nodules.

**Figure 4:**
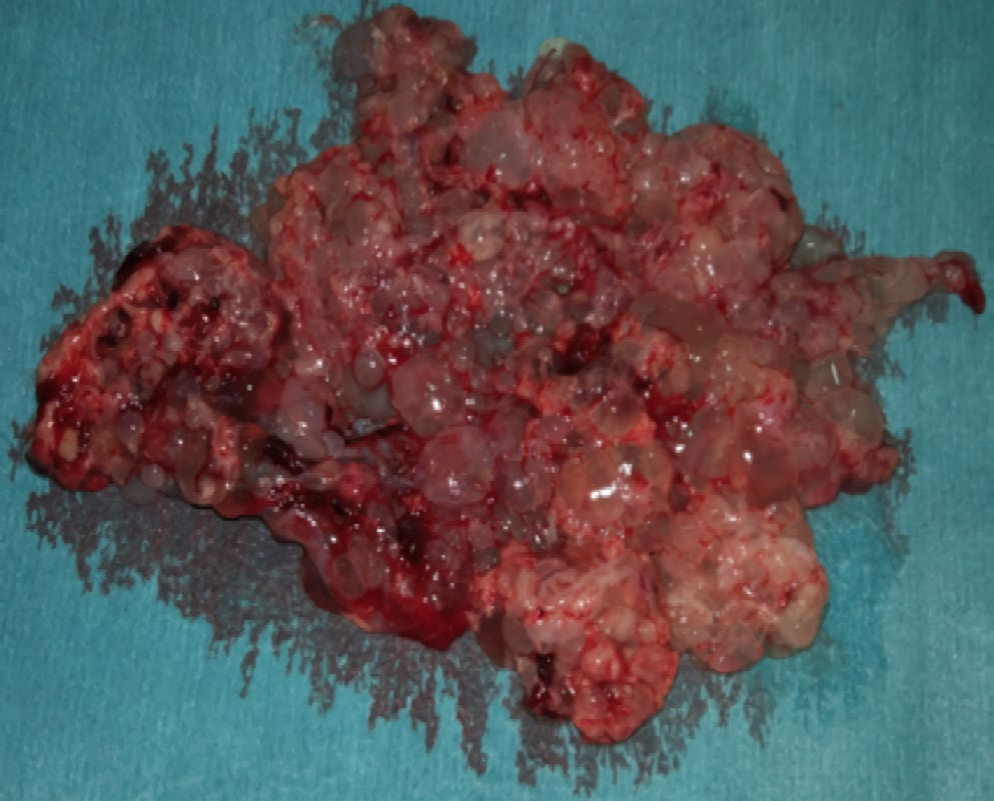
Uterine curettage aspiration

**Figure 5:**
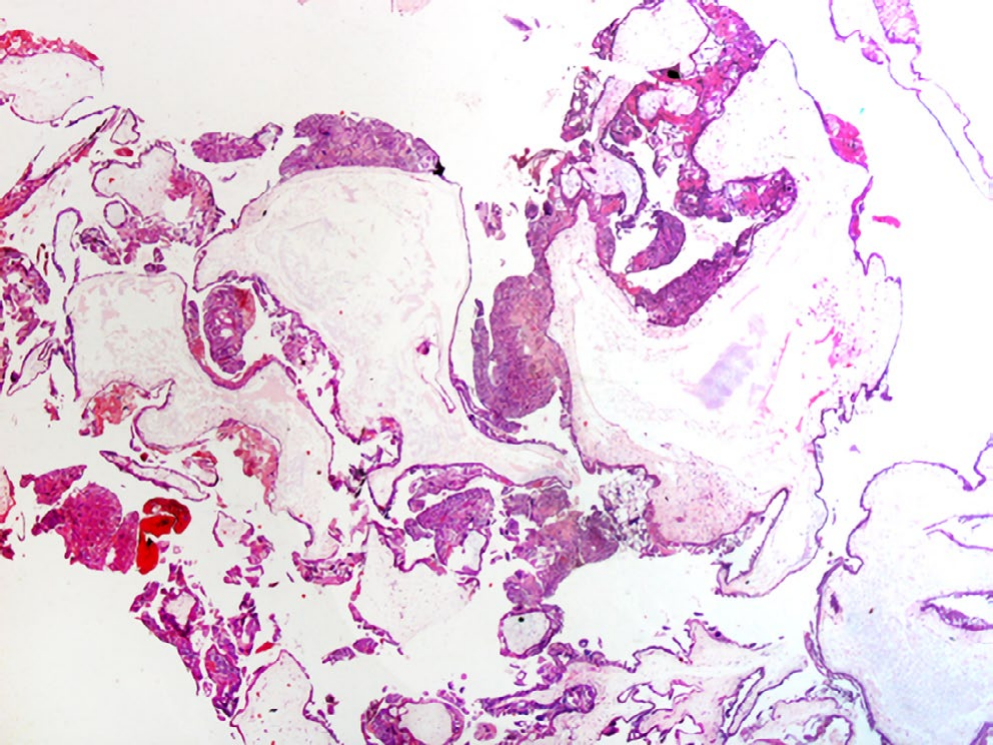
Complete hydatidiform mole. Diffuse villous enlargement cistern formation, hydropic changes, and trophoblastic hyperplasia

Transvaginal ultrasound showed an enlarged uterus with an intracavitary heterogeneous vacuolar image. BHCG was 684 180 mIU/mL. Suspecting GTD, uterine curettage aspiration was performed and confirmed the histological diagnosis of complete mole (Figures 4 and 5).

**Figure 6:**
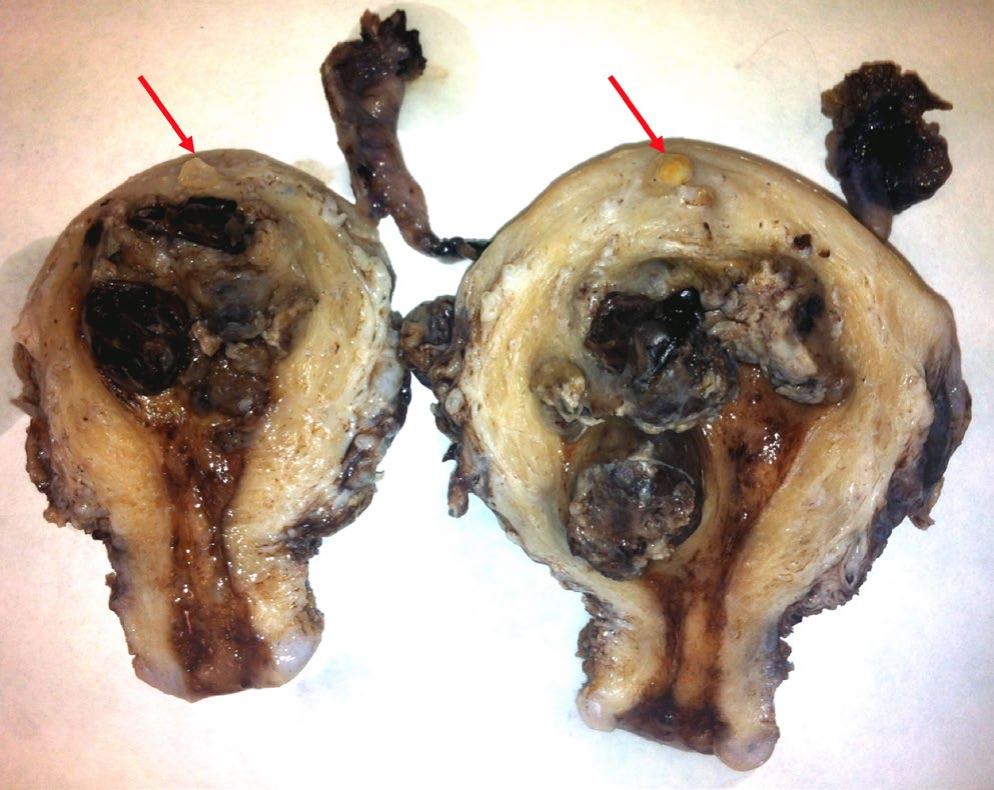
Piece of hysterectomy: Intracavitary persistent gestational trophoblastic tissue. Intramyometrial vesicles invasion (arrow)

Follow‐up monitoring BHCG was decided. One week after surgical evacuation, the patient reported abundant vaginal bleeding with a BHCG plateau of 14 000 mUI/mL. On gynecologic examination there were no vulvar, vaginal, or cervical lesions. The transvaginal ultrasound showed an irregular intracavitary image of 46 × 30 mm with positive vascularization suggesting myometrial invasion. Moreover, an increase in BHCG to 19 453 mIU/mL was detected. An urgent hysterectomy (Figure 6) was performed due to uncontrollable vaginal bleeding resulting in the patient being hemodynamically unstable with a blood pressure of 85/50 mm Hg, pulse rate of 120 bpm, hemoglobin: 7.2 g/dL, hematocrit: 22%, and transfusion of 2 units of packed red blood cells.

**Figure 7:**
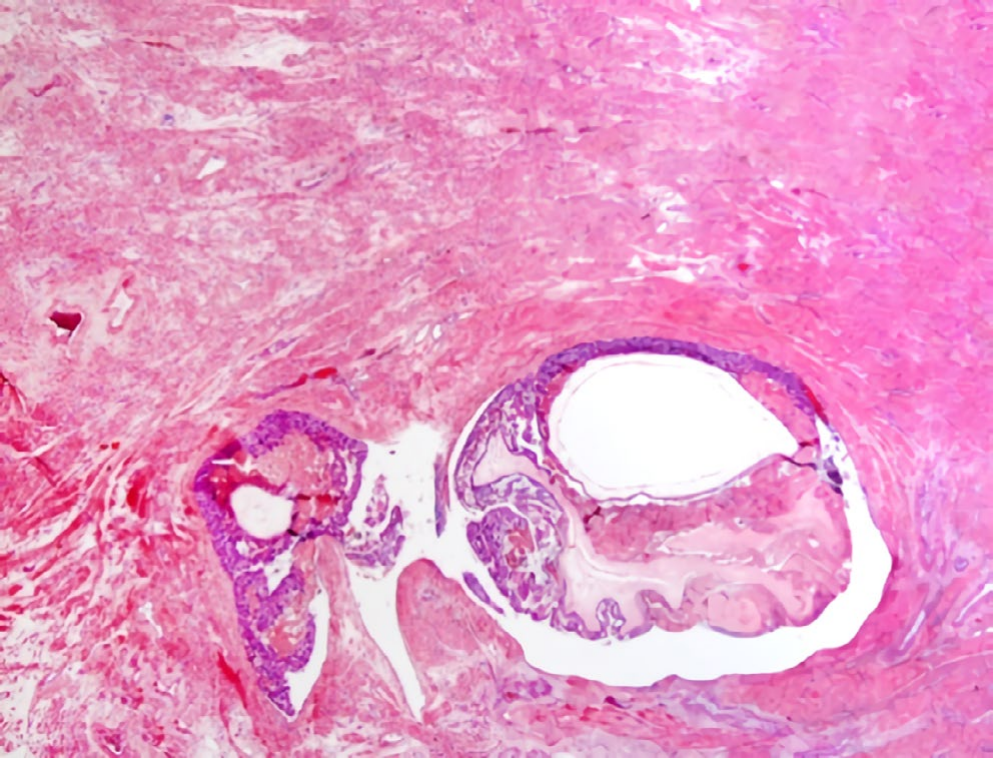
Invasive complete hydatidiform mole. Molar villi within the myometrium

**Figure 8:**
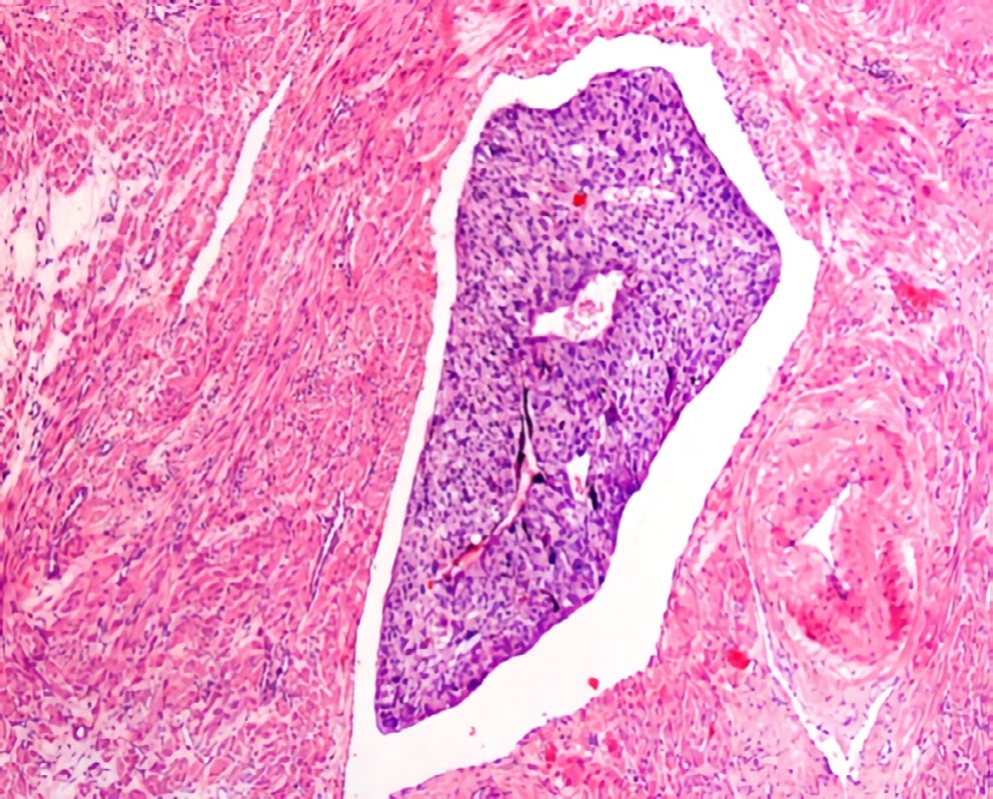
Detail of vascular invasion

Subsequently, the presence of myometrial invasion was confirmed histologically (Figures 7 and 8).

**Figure 9:**
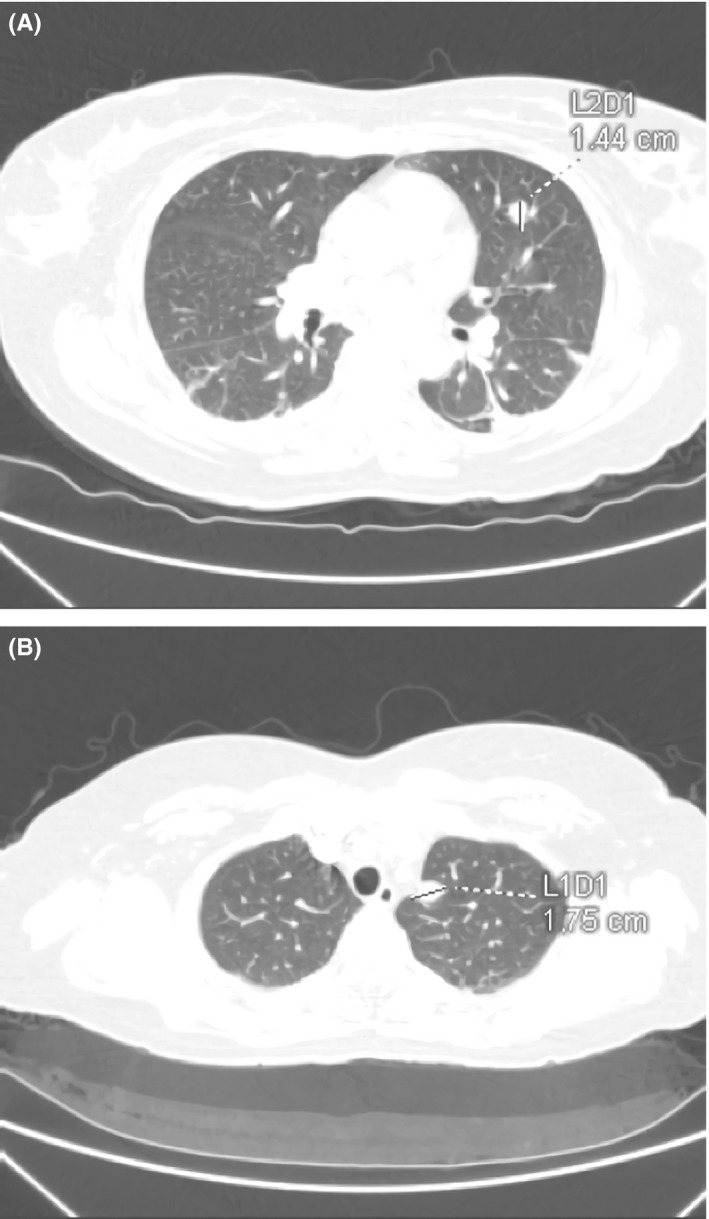
Thoracoabdominopelvic CT scan: Pulmonary nodules of 1.44 cm and 1.75 cm suggestive of pulmonary metastases

On the first day of admission, a thoracoabdominopelvic CT scan was requested, finding new bilateral pulmonary nodules suspected to be metastases (Figure 9). The brain MRI was normal.

**Figure 10:**
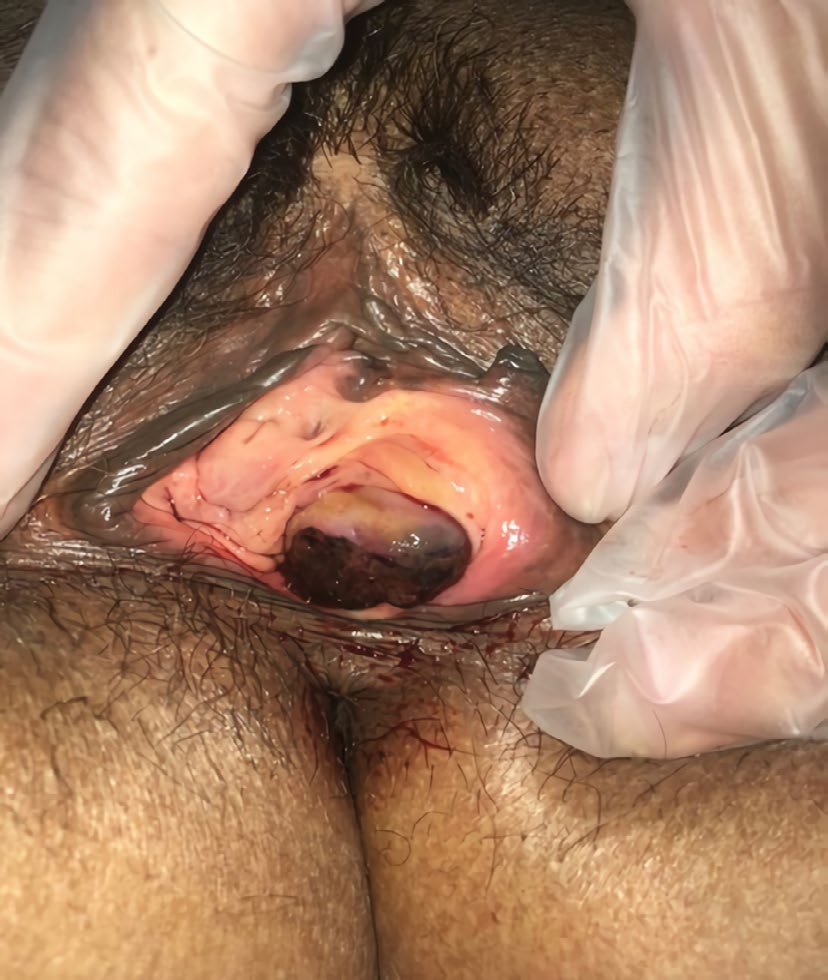
Vaginal metastases

Seven days after hysterectomy (fourteen days postmolar evacuation), she came back to the emergency room due to an increase in vaginal bleeding. A bleeding mucosal vaginal lesion of 2 cm suggested vaginal metastasis located in the paraurethral region adjacent to the introitus (Figure 10).

With a diagnosis of an invasive mole with pulmonary and vaginal spread, the patient was referred to the Oncology Department. The patient began chemotherapy with EMA‐CO (Etoposide + Methotrexate + Actinomycin‐Oncovin + Cyclophosphamide). After the third cycle, a significant decrease in BHCG with complete response of pulmonary and vaginal metastatic disease was achieved.

**Figure 11:**
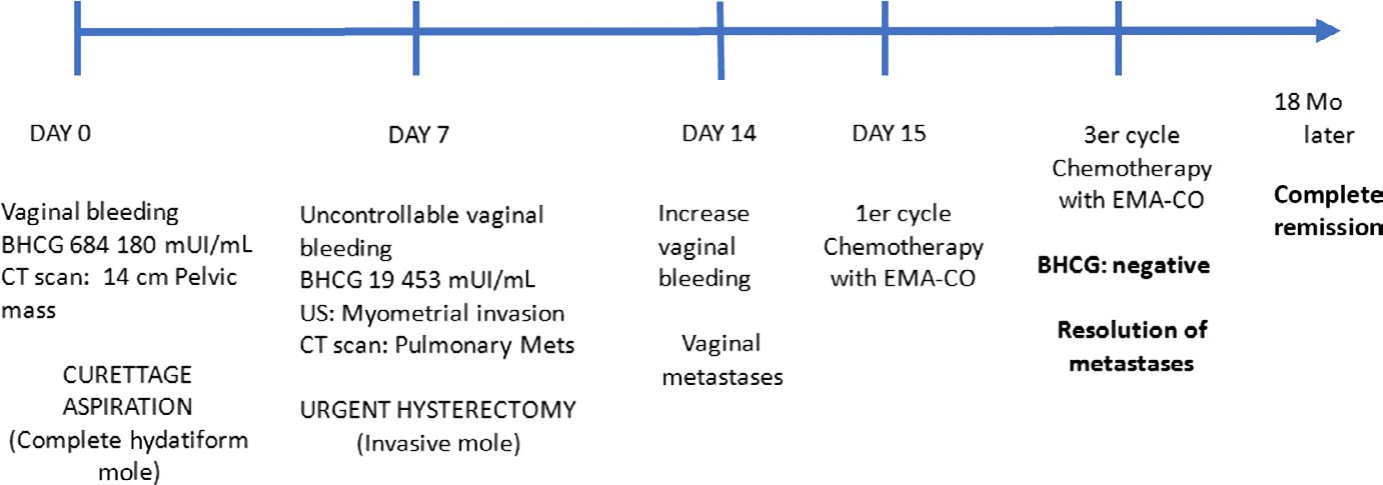
Chronological explanation of events

In summary, an initially bad prognosis with an extended metastatic disease reached a complete and stable response (Figure 11).

## 3 DISCUSSION

Hydatidiform mole is a benign tumor with malignant potential. Progression occurs in 15% of complete mole and 1.5% of partial mole.^9^ Moreover, term pregnancies carry a greater risk of progression than spontaneous abortion or a previous mole.^5^ In our case, the invasive mole progressed after the evacuation of a complete mole and previous obstetric history of 5 term pregnancies.

The classical medical presentation of GTD has decreased due to the early diagnosis in the screening in the first trimester. However, the risk of developing postmolar GTN remains without changes.^10^

Spontaneous presentation of an invasive mole is extremely rare. It is preceded by a hydatidiform mole in about 95% of cases with an interval of less than 6 months between the presentation of the case and the diagnosis of invasive mole. In our case, the progression to invasive mole was within 2 months from the evacuation of the complete mole. Follow‐up of such patients is essential for early detection of malignant trophoblastic tumors and to reduce mortality rate.^11^

Invasive mole usually occurs in women of reproductive age and is extremely rare in perimenopausal women. Only 5 cases of hydatidiform mole in postmenopausal women have been reported in literature since 2004.^12^ The pathogenesis of invasive mole in perimenopausal women is unclear, but it is believed that it may be due to immature spontaneous ovulation of oocytes leading to decreased fertility in perimenopausal patients and eventually to postmenopausal patients.

The most common locations for invasive mole metastases are the vagina, lungs, and brain, due to the invasion of molar tissue into the venous system. Other sites of metastases, including the epidural space and bladder, have been rarely reported.^13^

Seckl et al^6^ in 2000, locally invasive gestational trophoblastic neoplasia develops in 15% of patients and metastatic form in 4% of patients after evacuation of complete mole and infrequently after partial mole. In our case, metastases occurred in lungs and vagina, making an unusual presentation of invasive mole. Moreover, biopsy of metastasis is not recommended due to the risk of hemorrhage.^7^

Medical therapy is the best option and histological diagnosis is not mandatory for chemotherapy initiation. The diagnosis is confirmed using diagnostic imaging and serum BHCG.^7^

Hysterectomy may be required in cases of uncontrolled vaginal or intra‐abdominal bleeding (another option is the embolization of uterine vessels,^2^ but this is only considered if the patient is hemodynamically stable), resistance to chemotherapy or neoplastic gestational diseases. Surgical options could be a valid first‐line therapy mainly in women who do not wish to retain fertility, but it does not prevent the appearance of metastases.^14^ In our case, a hysterectomy was performed due to the life‐threatening hemorrhage and hemodynamic instability. However, it did not prevent the later appearance of the vaginal metastases.

Finally, in patients with an extended uterine tumor, hysterectomy could substantially reduce trophoblastic tumor burden and the number of chemotherapy cycles, thus reducing their toxicity.^14^ The final diagnosis of our patient was an invasive mole that progressed from a complete hydatidiform mole to eventual pulmonary and vaginal metastases. The patient is therefore classified as high risk (III:8) according to the FIGO staging system and WHO risk factor scoring (2) (FIGO III: disease in the lung. FIGO score 8: Age > 40 = **1 point**; Antecedent pregnancy: mole = **0 point**; Interval from mole and invasive mole: <4 months = **0 point**; BHCG pretreatment = >10^5^ = 684 180 mIU/mL = **4 points**; Largest tumor including uterus > 5 cm (14 cm) = **2 points**; Number of metastases identified: 3 (2 lungs and 1 vagina) = **1 point**; Site of metastases: Lungs = **0 points**; Tables 1 and 2) with a favorable clinical response to the established surgical and chemotherapy treatment. Our patient's serum BHCG levels gradually decreased to within normal range with the third cycle of chemotherapy; two additional cycles were given in order to reduce the risk of relapse. On the latest follow‐up, no evidence of disease has been observed after 18 months of completing chemotherapy.

**Table 1:** FIGO staging and classification for gestational trophoblastic neoplasia

FIGO stage	Description
I	Gestational trophoblastic tumors strictly confined to the uterine corpus
II	Gestational trophoblastic tumors extending to the adnexa or to the vagina, but limited to the genital structures
III	Gestational trophoblastic tumors extending to the lungs, with or without genital tract involvement
IV	All other metastatic sites

**Table 2:** WHO scoring system based on prognostic factors

WHO risk factor scoring with FIGO staging	0	1	2	4
Age	<40	>40	–	–
Antecedent pregnancy	Mole	Abortion	Term	
Interval from index pregnancy, mo	<4	4‐6	7‐12	>12
Pretreatment hCG mIU/mL	<10^3^	>10^3^‐10^4^	>10^4^‐10^5^	>10^5^
Largest tumor size including uterus, cm	–	3‐4	≥5	–
Site of metastases including uterus	Lung	Spleen, kidney	Gastrointestinal tract	Brain, liver
Number of metastases identified	–	1‐4	5‐8	>8
Previous failed chemotherapy	–	–	Single drug	Two or more drugs

We believe that our case will contribute to the literature with respect to the early detection of invasive moles and their complications. Although the first line of treatment is chemotherapy, we must consider the surgical option in patients who have uncontrollable hemorrhage and hemodynamic instability.

## CONFLICT OF INTEREST

None declared.

## AUTHOR CONTRIBUTION

MLC, GVJ: involved in data collection, study design, and manuscript writing. IF: provided us for histology part. GFJ: involved in critical revision. PAJ, AGM: are the chiefs of our Department and have a responsibility of all patients' outcome and paper publication.
